# EPR Monitoring of Oxygenation Levels in Tumors After Chlorophyllide-Based Photodynamic Therapy May Allow for Early Prediction of Treatment Outcome

**DOI:** 10.1007/s11307-023-01886-7

**Published:** 2024-01-31

**Authors:** Małgorzata Szczygieł, Barbara Kalinowska, Dariusz Szczygieł, Martyna Krzykawska-Serda, Leszek Fiedor, Aleksandra Anna Murzyn, Justyna Sopel, Zenon Matuszak, Martyna Elas

**Affiliations:** 1 Faculty of Biochemistry, Biophysics and Biotechnology, Jagiellonian University, Krakow, Poland; 2 Doctoral School of Exact and Natural Sciences, Faculty of Biochemistry, Biophysics and Biotechnology, Jagiellonian University, Krakow, Poland; 3 Department of Biophysics and Medical Physics, Faculty of Physics and Computer Science, AGH University of Science and Technology, Krakow, Poland

**Keywords:** Chlorophyllide, EPR Monitoring, Oxygenation, Photodynamic therapy (PDT), Melanoma

## Abstract

**Purpose:**

Molecular oxygen, besides a photosensitizer and light of appropriate wavelength, is one of the three factors necessary for photodynamic therapy (PDT). In tumor tissue, PDT leads to the killing of tumor cells, destruction of endothelial cells and vasculature collapse, and the induction of strong immune responses. All these effects may influence the oxygenation levels, but it is the vasculature changes that have the main impact on pO_2_. The purpose of our study was to monitor changes in tumor oxygenation after PDT and explore its significance for predicting long-term treatment response.

**Procedures:**

Electron paramagnetic resonance (EPR) spectroscopy enables direct, quantitative, and sequential measurements of partial pressure of oxygen (pO_2_) in the same animal. The levels of chlorophyll derived photosensitizers in tumor tissue were determined by transdermal emission measurements.

**Results:**

The noninvasive monitoring of pO_2_ in the tumor tissue after PDT showed that the higher ΔpO_2_ (pO_2_ after PDT minus pO_2_ before PDT), the greater the inhibition of tumor growth. ΔpO_2_ also correlated with higher levels of the photosensitizers in the tumor and with the occurrence of a severe edema/erythema after PDT.

**Conclusion:**

Monitoring of PDT-induced changes in tumor oxygenation is a valuable prognostic factor and could be also used to identify potentially resistant tumors, which is important in predicting long-term treatment response.

**Supplementary Information:**

The online version contains supplementary material available at 10.1007/s11307-023-01886-7.

## Introduction

The tumor microenvironment (TME) is a complex and dynamic system which interacts with tumor cells and defines the effects on tumor development and progression. TME differs significantly from healthy tissue microenvironment and, among others, is characterized by regions of reduced oxygen concentration and nutrient deprivation and contributes to uncontrolled proliferation and abnormal angiogenesis, which drives more resistance to anticancer therapies [1]. Poorly formed tumor blood vessels limit oxygen supply to the growing tumor, and the consumption of oxygen by the tumor cells causes an imbalance between oxygen consumption and oxygen supply [2]. Tumor tissue consumes a lot of energy due to the enhanced metabolism and intense cell proliferation. The supply of oxygen by the vasculature leads to tumor hypoxia early during tumor development due to high energy demand by quickly dividing cells.

Photodynamic therapy (PDT) of tumors leads three major effects: the killing of tumor cells, destruction of the vasculature, and induction of an immune response.

Tumor tissue oxygenation levels are dynamically changing during and after PDT, as a result of decreased oxygen delivery and consumption [3]. Measurements of oxygen before, during, and after PDT may provide important information relevant for predicting the treatment effect, determining the treatment regimen, or providing other treatments (e.g. radiotherapy), and understanding the processes occurring in the tissue after PDT [4, 5]. However, for PDT to be effective, efficient photosensitizers are needed. Promising photosensitizers are chlorophyllide derivatives, in particular chlorophyllide a (Chlide) and a zinc derivative of pheophorbide a (Zn-Pheide). This family of photosensitizers is characterized by low cytotoxicity, high efficiency in generating reactive oxygen species (ROS), and strong absorption of light in the visible part of the spectrum, even at low light doses, coinciding with the therapeutic window of human tissue, i.e., a spectral range where the light penetrates relatively deeply as it is not absorbed by endogenous skin pigments or water. This indicates a high photodynamic potential of these pigments. The *in vitro* and *in vivo* studies conducted so far have confirmed the high effectiveness of anticancer therapies which contribute not only to the inhibition of tumor growth but also to tumor regression [6–8].

EPR oximetry allows for direct measurements of partial pressure of oxygen (pO_2_) within the tissue, in the real time [9]. There are two types of paramagnetic oxygen sensors: soluble and particulate probes. Soluble probes measure product of molecular oxygen concentration and its diffusion coefficient, and particulate probes report pO_2_ at their immediate vicinity. Local measurement in conjunction with the non-toxicity, high stability in the tissue and a very low reactivity of particulate probes allows to perform multiple measurements of oxygen levels over a long period of time (several days) at the same tissue site [10]. Lithium phthalocyanine (LiPc) is one of the solid-state probes, in the form of water-insoluble microcrystals which can be implanted into the tissue, showing substantial EPR signal dependent on the oxygen concentration. LiPc has a single narrow EPR line that expands due to interaction with molecular oxygen. In the wide range of oxygen concentrations, this relationship is linear, including physiological concentrations [11, 12].

The presence of oxygen is one of the key factors determining the effectiveness of PDT. Hypoxia substantially reduces the effectiveness of the therapy due to the fact that molecular oxygen is involved in all types of photosensitized reactions. During the formation of reactive oxygen species in photochemical reactions, molecular oxygen consumption is proportional to the light power density [13, 14]. These changes occur within seconds, parallel to the irradiation of the tumor [14].

Decreased oxygen concentrations in the tumor may result from the damage to blood vessels during and after the treatment. Necrosis may develop in areas of nutrient and oxygen deficiency. Blood vessels may close off, dilate, or start leaking within a few minutes to hours after the treatment, and this can then either progress or regress. It has been shown that changes in oxygen levels in tumor tissue during and after PDT depend mainly on the type and dose of the photosensitizer, the interval between photosensitizer administration and irradiation, and the dose of light [14]. Depending on the PDT protocol, either a decrease or an increase of the oxygen level in the tumor immediately after irradiation can be observed [15]. The increase in pO_2_ may be due to the lower oxygen consumption by the damaged cells [16] or to the intensification of tumor blood perfusion and vessel extension, due to an increase of temperature caused by irradiation [17].

In the present study, we aimed to assess the effects of chlorophyllide-based PDT on tumor oxygenation and to find the relationship between pO_2_ in PDT-treated tumors and treatment efficacy.

## Materials and Methods

### Tumor Cell Culture

Cloudman S91 mouse melanoma cells, subline S91/I3 (American Type Culture Collection, USA), were cultured adherently in RPMI 1640 medium (Sigma-Aldrich Co., Steinheim, Germany) with 5% (v/v) fetal calf serum FCS (Gibco, Carlsbad, USA) and antibiotics: streptomycin (50 ng/ml) and penicillin (50 U/ml) at 80% humidity and 5% CO_2_ content.

### Animals and Tumor Inoculation

**Fig. 1:**
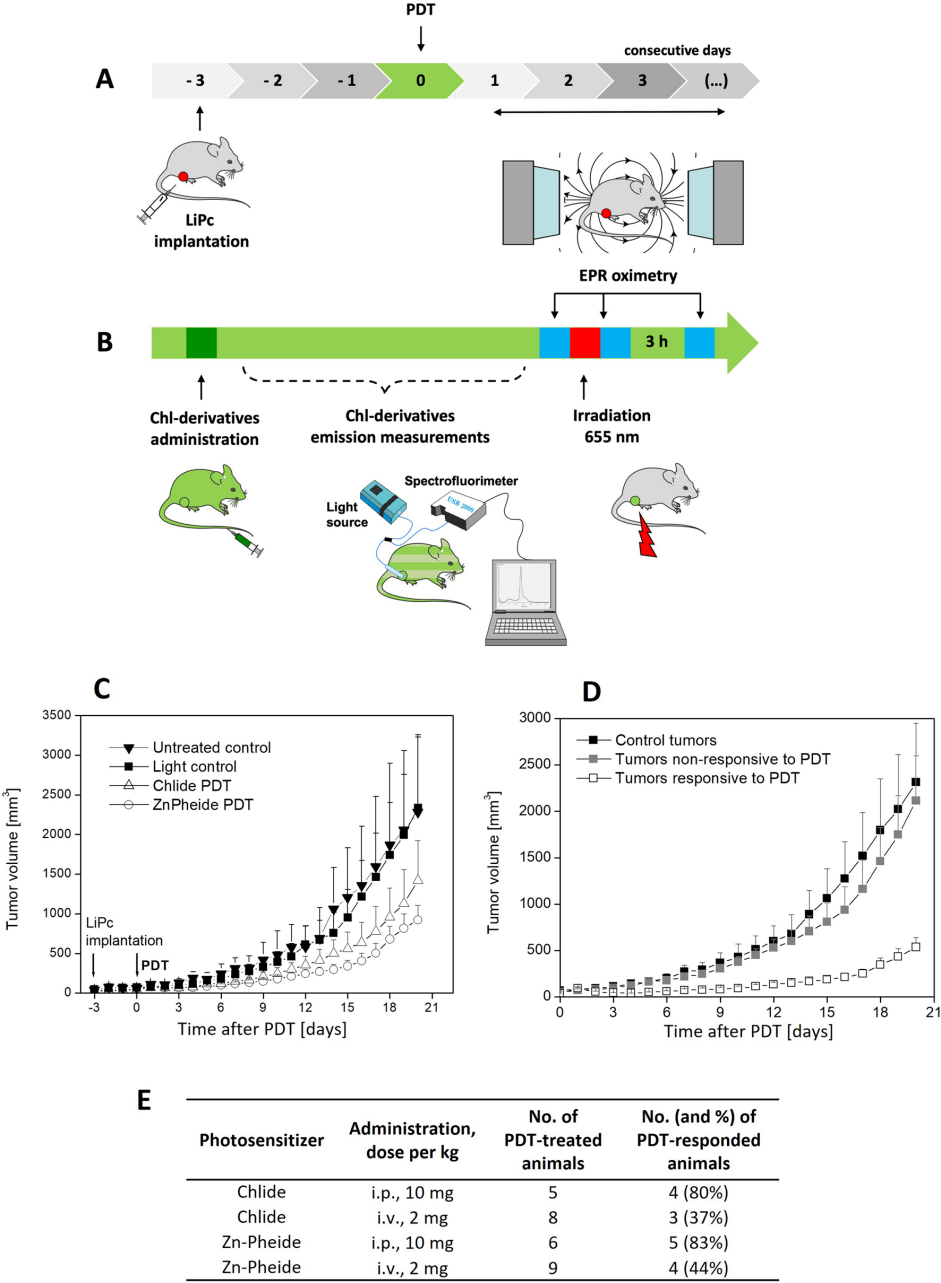
**A** Photodynamic therapy and related non-invasive measurements (pO_2_, photosensitizer level, blood perfusion, edema/erythema) were performed according to the scheme. **B** Plan of measurements on the day of PDT (details in “Materials and Methods”). **C** Kinetics of S91 tumors growth after chlorophyllide-based PDT. Chlide (*N* = 13) and Zn-Pheide (*N* = 15) effectiveness in PDT in comparison to control tumors (non-irradiated dark control, *N* = 9 and irradiated light control, *N* = 11). **D** comparison of tumor growth of responders (*N* = 16) and non-responders (*N* = 12) to either Chlide or Zn-Pheide PDT, and control tumors (*N* = 20). **E** Response of S91 tumors to chlorophyllide-based PDT in dependence on the photosensitizer dose and way of administration

Male DBA/2 mice (Institute of Animal Experimental and Clinical Medicine in Warsaw) of approximately 20–28 g, aged 3–8 months, were used. The study was carried out using male mice for better statistics and ethical reasons (fewer animals sacrificed), considering a smaller scatter of results than in the case of females. The hormonal cycle in the latter affects the biological results; moreover, melanoma tumors in mice may significantly differ in growth kinetics between the sexes [18]. Tumors were obtained by implanting 0.5 × 10^6^ S91 cells, suspended in 0.1 ml of PBS intradermally into the right hind leg of the animal. Solid tumors appeared approximately 10 days after implantation and were measured by caliper in three dimensions. Tumor volume (*V*) was estimated using the equation: *V* = (Π/6) *a* × *b* × *c*, where *a*, *b*, and *c* are the perpendicular diameters of the ellipsoid approximating the shape of the tumor. Tumor growth was monitored up to 20 days after PDT. The threshold of tumor progression was chosen as tumor volume higher than 400 mm^3^, as this is approximately 30% of the maximal tumor volume animals may carry and such a limit is often cited in the literature [14]. Photodynamic therapy and measurements (pO_2_, photosensitizer level, blood perfusion, edema/erythema) were performed according to the scheme included in the Fig. 1A, B. All animal experiment protocols were approved by First Local Ethical Committee for Experiments on Animals (Jagiellonian University, permission nos. 13/2010 and 132/2010).

### Probe Implantation and EPR Oximetry

Oxygen probe crystalline lithium phthalocyanine (LiPc) was a kind gift of Prof. H. Swartz (EPR Center for the Study of Viable Systems, The Geisel School of Medicine at Dartmouth, USA). The single LiPc microcrystal (weight about 0.1 mg, with size ranged from 0.1 to 0.8 mm) was implanted into the tumor with a sterile needle (0.6 mm diameter) after it had reached an average diameter of about 2.5 mm. Oxygen partial pressure values were read from the calibration curve (Figure S1 in the supplement). EPR measurements were performed using a Radiopan spectrometer, equipped with an S-band microwave bridge for 2.1 GHz (Jagmar, Poland) and a homemade surface coil at microwave frequency 2.12 GHz, microwave power 4.0 mW, modulation amplitude 0.08 Gs, modulation frequency 100 kHz, sweep time 512 s, time constant 300 ms, the number of field points 1024, and sweep range 5 Gs. EPR measurements were taken daily for 9 days from the time of implantation of the probe (implantation day = first day). During the measurements, the leg with the tumor was immobilized and placed in the vicinity of the surface coil. Probe implantation and all EPR measurements were performed under full anesthesia via a mixture of ketamine (Vetaketam, a dose of 90 mg/kg) and medetomidine (Cepetor, a dose of 0.1 mg/kg).

### Ultrasonography (USG)

Imaging was performed with an VEVO 2100 (VisualSonics, Toronto, ON, Canada) equipped with MS-550S transducer (32–56 MHz). Images of LiPc crystal localization have been done in B-mode.

### Preparation of Photosensitizers

Chlide was obtained via enzymatic hydrolysis of chlorophyll a and then purified chromatographically according to a previously described method [19]. Zn-Pheide was prepared via a direct metalation of pheophorbide a with Zn^2+^ acetate and then purified chromatographically as described previously [20].

### Photodynamic Therapy

Two derivatives of chlorophyll a were used: Chlide and Zn-Pheide (Figure S2); therapy was applied on the third day from LiPc implantation. In the appropriate groups, photosensitizers were administered intraperitoneally (i.p., 10 mg/kg of body weight) and intravenously through the tail vein (i.v., 2 mg/kg of body weight). PDT was performed after reaching the maximum level of the photosensitizer in the tumor tissue (approximately 1.5–4.5 h). The dose level of the photosensitizer in tumors was measured transdermally by fluorescence detection (Figure S2). The tumors were irradiated with a diode laser (Creotech, Warsaw, Poland) with a wavelength of 655 nm and a power density of 60 mW/cm^2^ for 25 min with a total administered dose of 100 J/cm^2^. Photodynamic therapy was performed under full anesthesia.

### Monitoring of Photosensitizer Level in Tumors *In Vivo*

The measurements were performed using a portable USB2000 spectrometer equipped with a QR200-7-UV–Vis Fiber Fluorescence Probe (Ocean Optics, USA). The administered photosensitizer was excited directly through the skin of the animal using the LS-450 light source, as described in [18, 21].

### Blood Perfusion Measurements

Measurements were performed with an LDPI (Laser Doppler Perfusion Imaging) Periscan system II (Perimed AB, Järfälla, Sweden) before, immediately after the PDT, 3 h after the PDT and then every 24 h for several consecutive days. Microcirculatory blood perfusion was measured in the tumor area, and in the same region in the contralateral leg without tumor, as described in [19, 22].

### Tumor Edema and Erythema Evaluation

Edema was expressed as tumor volume compared to pre-treatment tumor volume. Erythema was evaluated based on a four-grade scale; where 0 means no erythema; 1, minor; 2, moderate; and 3, intense erythema.

### Software and Analysis

Fitting of the experimental spectra to extract the linewidth was carried out using EPR Fitting Software v3.0.2 K Krakow (Benjamin B. Williams, Tom Matthews, Dartmouth EPR Center, USA). Analysis of the results was carried out using programs Excel 2003, Origin 7.5 and Statistica 5.1. Graphic elements of some figures were created using Servier Medical Art templates. The results are presented as mean ± SE; the statistical significance of differences between the means was assessed using the Student *t*-test.

## Results

### Tumors Response to PDT

In most animals after PDT, there was a clear response to treatment manifested by inhibiting or slowing down tumor growth (Fig. 1C, D). The tumor response to PDT was dependent on the photosensitizer dose and the protocol applied. The intraperitoneal administration allows using a higher dose of the compound (10 mg/kg) than intravenous (2 mg/kg), which resulted in higher number of responders to therapy regimen (Fig. 1E).

The comparison for all the experimental groups is shown in Fig. 1C. All the PDT-treated animals were divided into responders and non-responders, regardless of the photosensitizer applied or the route of the treatment; the growth kinetics of responding vs. not responding tumors is shown in Fig. 1D. The tumor size was used as a metrics. The treatment caused tumor swelling, and the response assessment was feasible only after the swelling had subsided, i.e., 5 days after irradiation (Fig. 4C). Some tumors showed a marked growth slowdown 5 days after PDT, and the increase in their volume increased only slightly, by less than 100% (tumors responding to PDT: Δ*V*(5 days) < 100%). However, in the case of the remaining PDT tumors, the increase in volume after 5 days after PDT was strong and similar to that in control tumors, and ranged from 100 to 500% compared to the volume before irradiation (tumors non-responding to PDT: Δ*V*(5 days) > 100%). The responders revealed a significant inhibition in comparison to control tumors (Fig. 1D). The response to the treatment also depended on the type of central metal in macrocycle ring of photosensitizer. Replacement of the central Mg^2+^ ion with Zn^2+^ ion led to a higher number of responses to PDT and a slower rate of tumor growth (Fig. 1C, E).

### Partial Pressure of Oxygen in Tumors

**Fig. 2:**
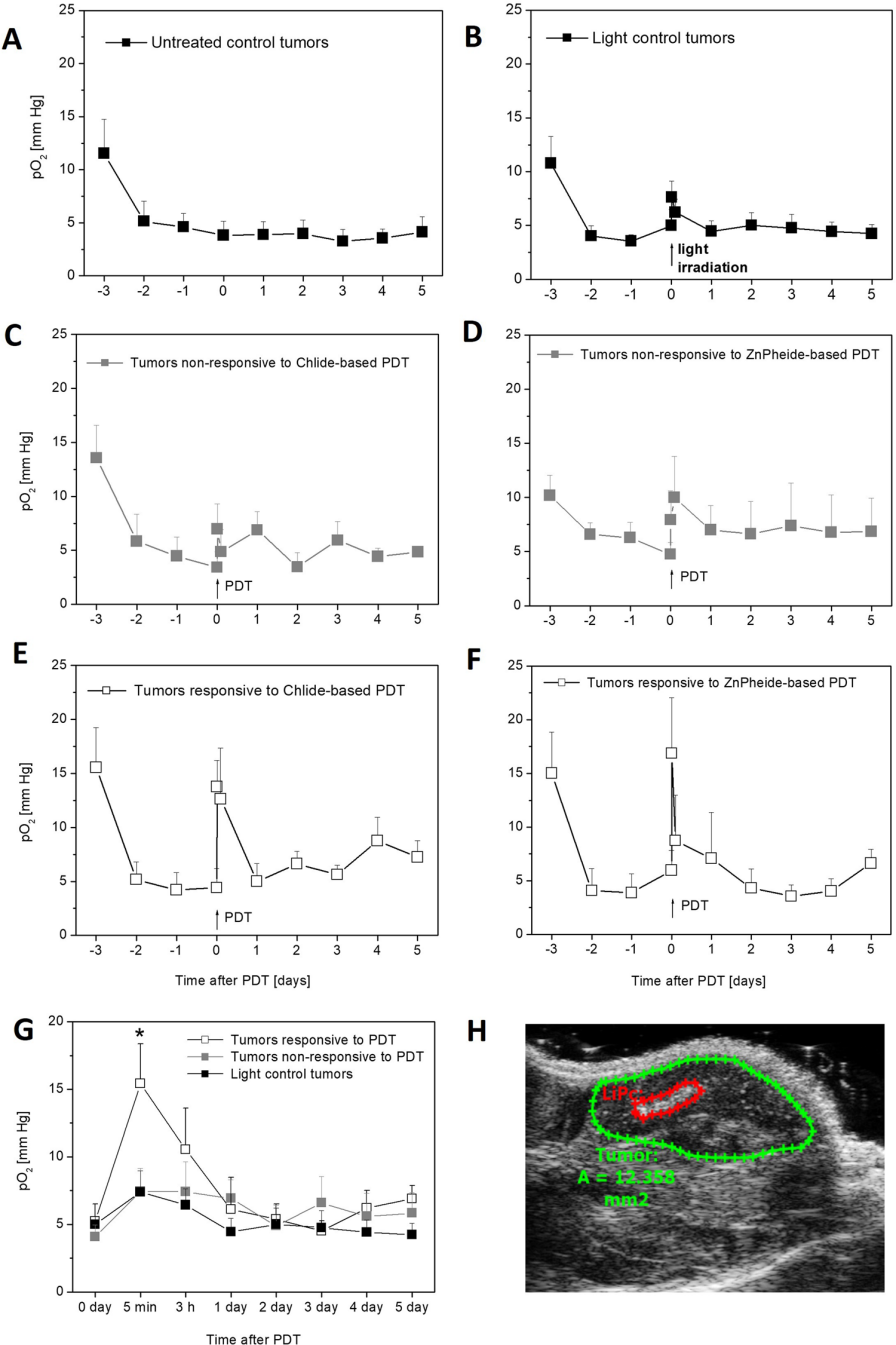
Partial pressure of oxygen (pO_2_) in S91 tumors before and after chlorophyllide-based PDT. **A** Control tumors (untreated); **B** control tumors (irradiated without photosensitizer); **C** tumors non-responsive to Chlide-PDT; **D** tumors non-responsive to Zn-Pheide PDT; **E t**umors responsive to Chlide-PDT; **F** tumors responsive to Zn-Pheide PDT. **G** pO_2_ in tumors after PDT (Chlide and Zn-Pheide) in comparison to light control tumors; summary of data presented in A–F. *Statistical significance of difference between the means *p* < 0.05. **H** LiPc in tumor visualized by USG

In the non-irradiated control tumors at the day of LiPc implantation, the EPR signal decreases and in the following days, it is quickly stabilized at a relatively constant level, corresponding to the mean pO_2_ value of about 4–5 mmHg (Fig. 2A). The irradiated control tumors showed similar results (Fig. 2B); however, the irradiation itself led to slight short-time increase in pO_2_ in the tumor by about 50% (on the average from 5.0 to 7.6 mmHg, statistically not significant). Immediately after the light exposure, pO_2_ fell down, and already by the next day, it returned to the initial values and remained at this level for the following 5 days (Fig. 2B).

In the tumors subjected to PDT, very similar changes of pO_2_ after PDT were observed for both photosensitizers (Fig. 2 C, D, E, and F), and therefore, a collective analysis was performed with respect to light irradiated control tumors (Fig. 2G). Before irradiation, the mean levels of pO_2_ were similar in both the tumors subjected to PDT and the light control tumors (respectively, 4.7 and 5.0 mmHg). Already 5 min after PDT, the pO_2_ in the tumors increased from 4.7 to 11.6 mmHg, i.e., 150%. This effect is statistically significant if compared to the light irradiated control (pO_2_ increase was 6.9 mmHg in the PDT group versus 2.6 mmHg in the control group, *p* = 0.023). Afterwards, the pO_2_ value began to fall in tumors subjected to PDT, and on the following day, it returned to the initial level.

The tumors responsive to PDT showed an up to threefold increase in pO_2_ (on the average from 5.2 to 15.4 mmHg) (Fig. 2E, F), while pO_2_ in the non-responsive tumors only slightly increased after irradiation, from 4.1 to 7.4 mmHg (Fig. 2C, D), similar to the effect observed in the irradiated control tumors (5.0 to 7.6 mmHg) (Fig. 2B). During the irradiation of the PDT-responsive tumors, pO_2_ increase was about 10.2 mmHg and was statistically higher, in comparison both to tumors without response to PDT (pO_2_ increase after irradiation only 3.4 mmHg, *p* = 0.01) and to irradiated tumor controls (pO_2_ increase after irradiation only 2.6 mmHg, *p* = 0.005) (Fig. 2G).

The responsive tumors before the treatments were not statistically different in pO_2_ levels from both the tumors without response to PDT and control tumors. In the tumors with response, 1 day after irradiation, the pO_2_ level falls to the initial level and remains such during the following 5 days (Fig. 2G).

**Fig. 3:**
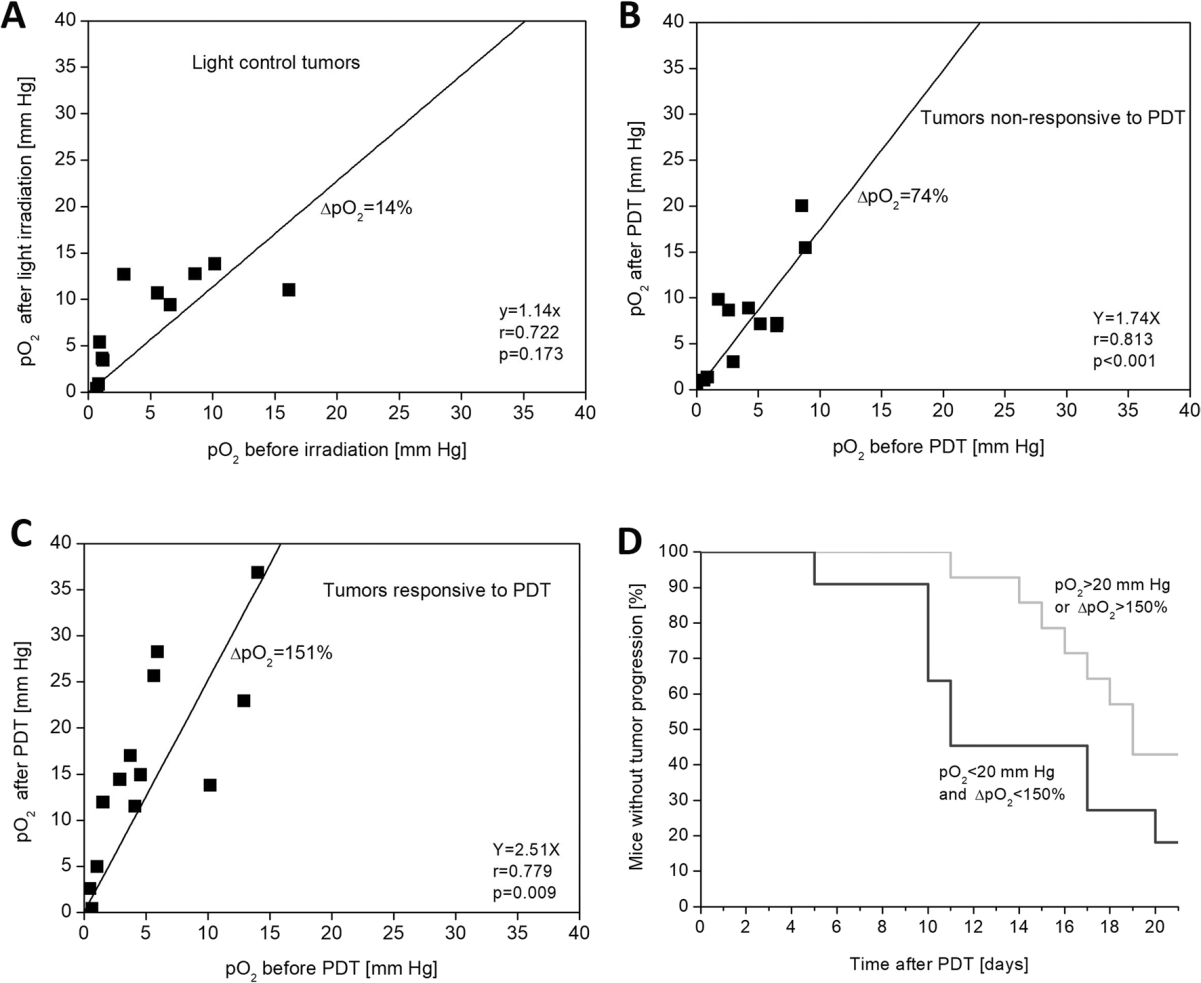
Correlation of pO_2_ level in the tumors at 5 min before and 5 min after irradiation. Points were fitted with the line passing through the (0.0) point. The slope of the curve represents an average of relative increase of pO_2_ in tumors after chlorophyllide-based PDT (Chlide and ZnPheide). Graphs respectively for **A** light-control tumors; **B** non-responsive tumors; **C** responsive tumors;﻿ **D** Partial pressure of oxygen as a prognostic factor of tumors response to chlorophyllide-based photodynamic therapy. Better response was seen in tumors with pO_2_ > 20 mm Hg after PDT at 5 min or percentage increase of partial pressure of oxygen ∆pO_2_ > 150% after PDT at 5 min. Median ΔpO_2_ 48% (2.77 mmHg) in light control tumors; 61% (1.29 mmHg) in tumors non-responsive to PDT; 348% (9.95 mmHg) in tumors responsive to PDT

In PDT-treated tumors, both responding and non-responding to the therapy, pO_2_ after PDT was generally increasing; however, pO_2_ increase was clearly correlated with the pre-exposure level. The correlation coefficients were *r* = 0.78 (*p* < 0.01) and *r* = 0.81 (*p* < 0.001), respectively (Fig. 3B, C). In the light-control tumors, no such correlation was observed, as coefficients were *r* = 0.72 and *p* = 0.17 (Fig. 3A). Linear fitting showed that the highest slope was observed in tumors responding to PDT. The values of the slopes indicate that the level of post-irradiation tumor pO_2_ in the tissue increases on average 2.5 times in tumors responding to PDT, 1.7 times in tumors non-responding (Fig. 3A, B, C).

The scatter of pO_2_ values prior to irradiation was similar, ranging from 1 to 15 mmHg in all three experimental groups. Immediately after irradiation, pO_2_ ranged 1–15 mmHg in light control tumors, 1–20 mmHg in tumors non-responsive to PDT, but it was much higher, 1–40 mmHg, in tumors responsive to PDT. In 30% of responsive tumors, pO_2_ achieved level 20–40 mmHg, while in non-responsive tumors and light control tumors, pO_2_ level never exceeded 20 mmHg immediately after PDT (Fig. 3 A, B, and C).

These results indicate that pO_2_ tumor level after chlorophyllide-based PDT with 660 nm light predicts the effectiveness of the therapy. Reaching pO_2_ > 20 mmHg or more than 2.5-fold increase (ΔpO_2_ > 150%) immediately after PDT correlates with tumor growth inhibition. Median time to tumor progression was 11 days in the group with unfavorable pO_2_ (pO_2_ < 20 mmHg and ΔpO_2_ < 150%), whereas in the group with favorable pO_2_ (pO_2_ > 20 mmHg or ΔpO_2_ > 150%), median time to tumor progression was 19 days, an increase of 70% (test log-rank p < 0.05) (Fig. 3D). For the positive prognosis categorization: 31% of tumors show pO_2_ > 20 mmHg (*N* = 4/13), 77% of tumors show ΔpO_2_ > 150% (*N* = 10/13), and 85% show pO_2_ > 20 mmHg or ΔpO_2_ > 150% (*N* = 11/13).

### Photosensitizers in Tumors

The efficacy of PDT depends strongly on the local concentration of photosensitizer in the tissue. As the quantity of photosensitizers Chlide and its metalo-substituted analogue Zn-Pheide can be measured fluorometrically, we have determined the level of photosensitizers in S91 tumors prior to light irradiation and correlated with tumor response to the photodynamic therapy and changes in tumor oxygenation.

**Fig. 4:**
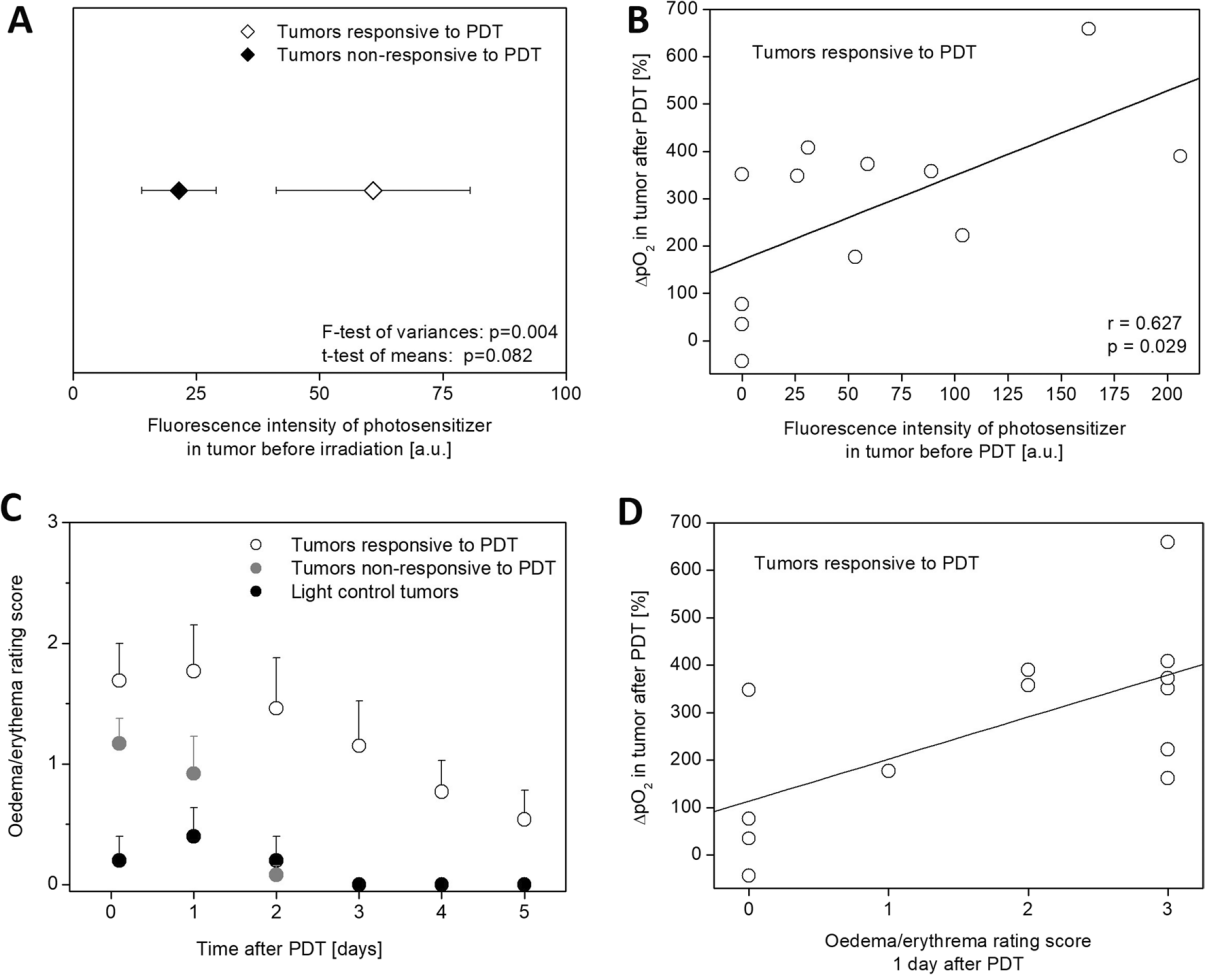
**A** The influence of the photosensitizer level in tumors on the response to PDT. Tumors responsive to the therapy had an average of about threefold higher fluorescence intensity of the photosensitizer than the non-responsive tumors. **B** Correlation of the photosensitizer level in tumors prior to irradiation (5 min) with percentage increase of pO_2_ after irradiation (5 min). The higher the level of the photosensitizer in the tumor prior to irradiation, the stronger the relative increase of pO_2_ in the tumor after irradiation. Revealed correlation is statistically significant, *p* < 0.05. **C** Evaluation of edema and erythema in tumors after chlorophyllide-based PDT (Chlide and Zn-Pheide). Differences in edema/erythema rating score between tumors responsive to PDT and light control tumors are statistically significant at each analyzed time point after irradiation (from 3 h to day 5). **D** Correlation between percentage increase of pO_2_ after PDT (5 min) and edema/erythema rating score after PDT (1 day)

Tumors which responded to PDT were characterized, on the average, by about threefold higher fluorescence intensity than the non-responsive tumors (Fig. 4A). In the responsive tumors, a statistically significant correlation between the level of the photosensitizer before irradiation (5 min) and the increase in tumor oxygenation after irradiation (5 min) was observed. The higher the level of the photosensitizer in the tumor prior to irradiation, the stronger the percentage increase of pO_2_ in the tumor after irradiation, *r* = 0.63, *p* < *0.*05 (Fig. 4B). In contrast, no such a correlation was seen in the non-responsive tumors (*r* =  − 0.23, *p* = 0.51).

### Edema and Erythema Around Tumors

After PDT, in some animals, the presence of edema or a strong redness around the tumor was observed, which persisted for several days. Therefore, animals were evaluated for intensity of these changes by using the three-step scale described in the “Materials and Methods” section. The results of this evaluation are presented in Fig. 4C.

After irradiation of control tumors, in most cases, there was no redness or swelling around the tumor. In a few cases, these changes occurred after irradiation, but the intensity was weak, and the effect lasted about 1 day. In the majority of tumors subjected to PDT, after several minutes of irradiation, the swelling began to appear, and usually after 3 h was already very noticeable. The swelling was strong in the well responding tumors, often with the appearance of erythema and lasted for up to 5 days. Also, in some cases, few days after the PDT, skin discoloration over the tumor was observed, which sometimes turned into scab. In the non-responsive tumors swelling or redness also appeared but were less severe and did not last longer than one day (Fig. 4C). Incidence of edema and its intensity in the responding tumors were correlated with the subsequent relative increase of pO_2_ after photodynamic therapy (Fig. 4D).

### Superficial Blood Flow in Tumors

**Fig. 5:**
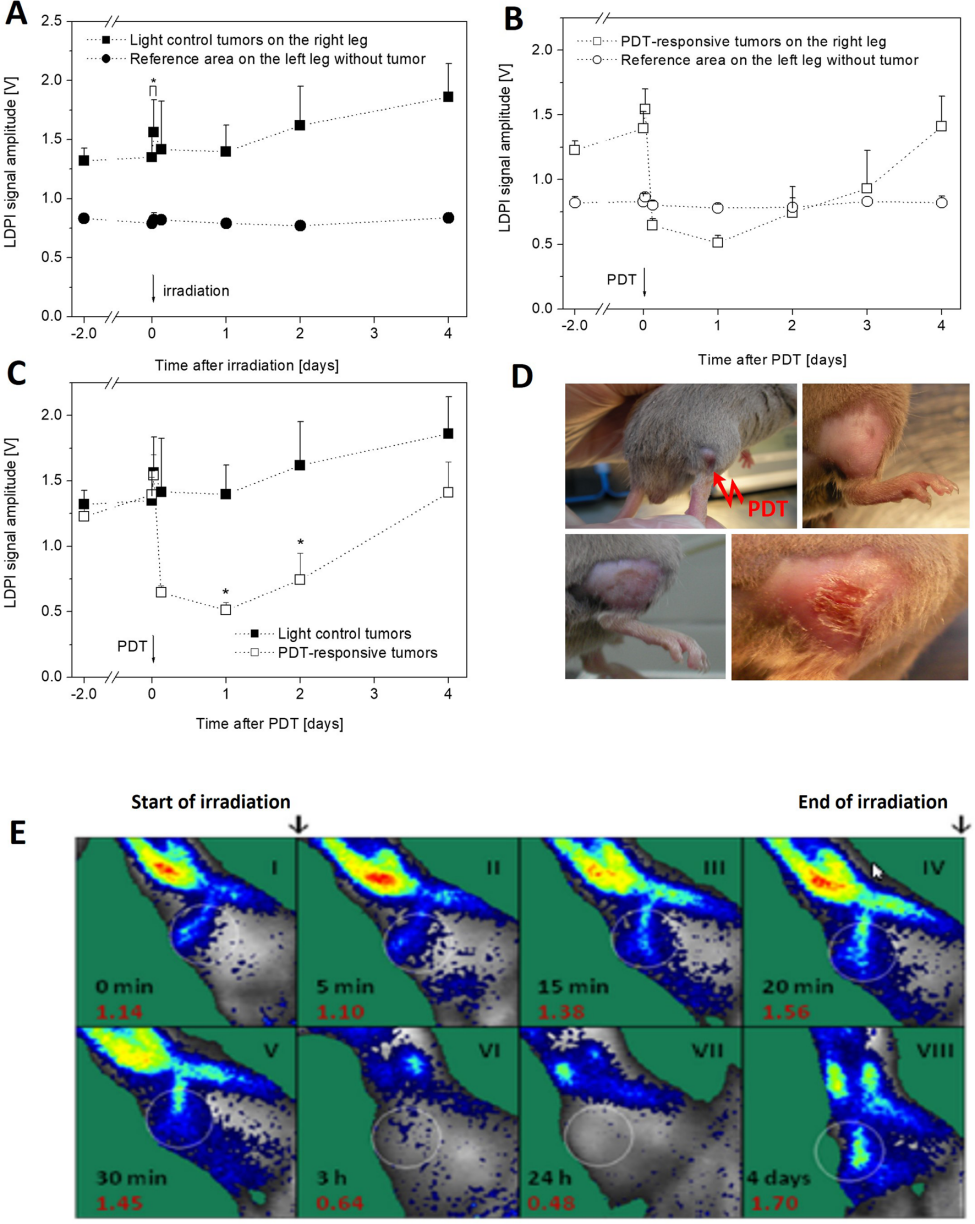
Superficial blood flow in S91 tumors as measured signal of laser Doppler perfusion imaging (LDPI). **A** Light control tumors (*N* = 5). *Statistically significant difference of superficial blood flow in the tumors just before and after irradiation (paired *t*-test: *p* < 0.05). **B** Tumors responsive to the PDT (*N* = 6). **C** Light control tumors versus PDT responsive tumors. **D** Representative images of tumors in sequence: just before PDT, day after, 4 days, week after. **E** Images of LDPI signal in the tumor with the strongest response to the Zn-Pheide PDT. The images were recorded just before (I), during (II, III, IV), and after irradiation (V, VI, VII, VIII). White circles indicate the area of the tumor localization, where the mean signal amplitude was calculated. The values of LDPI signal amplitude in the tumor (red) and time from the beginning of tumor irradiation (black) are shown in each image

Laser Doppler perfusion imaging is a technique used to evaluate the blood flow in the surface part of the tissue [20]. Irradiation of control tumors (light control, no photosensitizer given) using the 655 nm laser in all cases resulted in sharp increase of the surface tumor blood flow (*p* < 0.02). After 3 h, the intensity of the tumor blood flow returned to a level close to that before irradiation; however, in the subsequent 4 days in these tumors, a gradual increase of the surface blood flow together with the growth of the tumor mass were observed. Reference measurement performed in the second leg (without tumor and not irradiated) did not reveal that the process of irradiating a tumor and also tumor growth significantly change the flow rate of blood in the contralateral leg (Fig. 5A).

In the tumors responsive to PDT, a rapid increase of surface blood flow immediately after irradiation was observed (Fig. 5B); however, this increase was not greater than the corresponding flow rise in the irradiated control tumors (Fig. 5 A, E). Then, the surface blood flow quickly decreased, and after 3 h from the irradiation, LDPI signal amplitude was much lower than before irradiation. Minimum of the signal observed at the surface of tumors was recorded 1 day after irradiation (Fig. 5C, E), when maximum swelling was observed (Figs. 4C and 5D), and for the next 3 days, the signal gradually increased, reaching on the day 4 a level similar to that before irradiation. Despite very significant changes in surface blood flow in tumors responding to PDT, there were no changes in the reference area on the contralateral leg (Fig. 5B).

After the irradiation of tumors, the increase of blood flow was seen not only at the surface of tumors, but also on the surface of normal tissues surrounding the tumor (Fig. 5C).

## Discussion

### ΔpO_2_ as a Predictive Parameter of PDT with Chlorophyll Derivatives

Oximetry measurements in the tumors treated with photodynamic therapy have shown that both the use of Chlide and of its [Zn]-analog elicited treatment response preceded by a sudden strong increase of pO_2_ in the tumor, observed immediately after the light irradiation. In the next hours, pO_2_ returned to the pre-irradiation level. In the case of tumors that did not respond to the therapy and also light control tumors, the increase of pO_2_ was definitely weaker (Fig. 2).

Chlorophyll derivatives during irradiation efficiently transfer excitation energy to oxygen molecules, generating highly reactive oxygen species [7], leading to oxygen consumption in the tissue [23]. This can generate reactive hyperemia involving a rapid intensification of blood flow in order to reduce the hypoxic tissue, which leads to a short-lived and temporary increase in oxygenation [22, 24].

The average values of pO_2_ measured just before the irradiation were at similar levels in the groups of tumors responsive and non-responsive to therapy and in the control irradiated tumors (Fig. 2G). In the context of the literature data of oxygen mechanism of photodynamic action caused by chlorophyll derivatives [8], it was reasonable to assume that the therapeutic effect should be stronger in tumors with higher initial oxygenation [25]; therefore, this result indicates that the relatively low level of tumor oxygenation before irradiation had only a minor impact for the effect of the PDT.

Single-point EPR oximetry using LiPc provides an estimate of pO_2_ for a particular area where the oximetry probe is located. Due to the tumor heterogeneity, poorly or well-oxygenated tumor areas are present, and the crystalline probe was located randomly, revealing changes of pO_2_ in its surroundings. As the tumor grows, the pO_2_ values measured by the LiPc probe reflect the local oxygenation level of a given area of the tumor rather than the entire tumor. The results in Fig. 3 show that PDT using chlorophyll derivatives caused a proportional increase in pO_2_ in tumors. Low pO_2_ values in tumors before PDT are generally accompanied by small absolute increases in pO_2_ (mmHg) in tumors after PDT, while high pO_2_ values in tumors before PDT are generally accompanied by large absolute increases in pO_2_ (mmHg) in tumors after PDT. This was particularly visible in tumors responding well to PDT therapy (Fig. 3C). The data can be interpreted that in poorly vascularized tumor/tumor area (low pO_2_), there may be little change in the absolute pO_2_ value during PDT, whereas in well-vascularized tumor/tumor area (high pO_2_), large absolute changes in pO_2_ during PDT may occur. This is why the tumors responsive to the photodynamic therapy showed a wide range the obtained pO_2_ values after irradiation, from 1 to 40 mm Hg (Fig. 3C). Therefore, among the analyzed oxygenation parameters, the best predictor of tumor response to photodynamic therapy with chlorophyll derivatives is the relative increase in partial pressure of oxygen in the tumor between the start and end of irradiation (ΔpO_2_). In responsive tumors, the relative increase in partial pressure of oxygen ΔpO_2_ after the end of irradiation generally exceeded 150%, while in non-responsive tumors, this occurred only sporadically (Fig. 3).

In addition, after irradiation of the tumors, areas with a pO_2_ above 20 mm Hg were observed only in responsive tumors (Fig. 3). The combination of both predictive parameters, Δ pO_2_ > 150% and pO_2_ > 20 mm Hg, allows, in 85% of cases, to correctly identify tumors that will respond to PDT with chlorophyll derivatives. In animals with a positive predictor, the median time to tumor progression was increased by more than 70% (Fig. 3D).

### ΔpO_2_ Correlates with the Level of the Photosensitizer

The results of this study have shown that in PDT-responsive tumors, the relative increase in partial pressure of oxygen just after irradiation (ΔpO_2_) correlates with photosensitizer levels in tumors just before irradiation. The higher the photosensitizer concentration, the higher the ΔpO_2_ (Fig. 4B). This may suggest that a high level of the photosensitizer in the tumor leads to a strong photodynamic effect in it (Fig. 4A). Also, a tumor with better vascularity is more efficiently penetrated by both the photosensitizer and oxygen [26].

However, the level of the photosensitizer did not correlate with the pO_2_ measured before irradiation, but only with the relative increase in pO_2_ after irradiation (∆pO_2_). Obtaining a high level of a chlorophyll-based photosensitizer in the tumor is a necessary condition for inducing a strong increase in pO_2_ after irradiation. Developed vascular network may promote better photosensitizer penetration into the tumor [26], and photodynamic reactions occur more efficiently, as measured by fluorimetry *in vivo*; responsive tumors had about threefold higher photosensitizer levels before irradiation than non-responsive tumors (Fig. 4A), which, through high oxygen consumption, forces an increase in blood flow in a well-vascularized tumor, which consequently leads to rapid and intense, but short-lived increase in pO_2_ [22]. After the irradiation is completed, partial pressure of oxygen slowly returns to its pre-PDT equilibrium state.

Our results can also be explained by an alternative mechanism. Higher levels of the photosensitizer lead to a rapid necrotic cell death after PDT﻿. The results of our study show that the response to the therapy correlates with the development of severe swelling and redness of the tumor, often immediately after irradiation, followed by the formation of necrotic lesions in the tumor (Fig. 4C). This leads to a sudden drop in oxygen consumption by the tumor cells. At the same time, irradiation of tumors alone increases blood flow and oxygen transport to the tumor, as indicated by the results of blood flow and pO_2_ measurements in irradiated control tumors (Figs. 2B and 5A). The increased blood flow and reduced oxygen consumption by tumor cells results in a strong peak in pO_2_ in tumors, which after the end of irradiation slowly returns, within hours, to the state of equilibrium before PDT.

The results of LDPI measurements of superficial blood flow in tumors show that the increase in blood flow in responsive tumors was similar or only slightly higher than in irradiated control tumors (Fig. 5). If the former mechanism (reactive hyperemia) were to be predominant, the increase in blood flow after irradiation should be more intense in PDT-responsive tumors. The observed greater increase in pO_2_, however, with a similar increase in blood flow after irradiation in responsive tumors compared to light control tumors, indicates that the latter mechanism is more likely or dominant.

### pO_2_ and the Photosensitizer Type

Both the use of Chlide and Zn-Pheide during PDT led to an increase in tumor pO_2_ after irradiation, but Zn-Pheide resulted in a slightly higher peak pO_2_ and stronger tumor response (Figs. 1A and 2C, D). The more photodynamically effective the chlorophyll derivative, the stronger pO_2_ peak induced in tumors *in vivo* after photodynamic therapy. Interestingly, in similar experiments in S91 tumors on PDT with bacteriochlorin F_2_Bmet, which is currently being investigated in clinical trials (NCT02070432) [27], an immediate decrease in pO_2_ after irradiation was shown [22]. In addition, a high degree of inhibition of tumor growth after PDT with F_2_BMet correlates with the long-term state of hypoxia in them [22]. Chlorophyll metal derivatives and bacteriochlorin F_2_BMet have different effects on pO_2_ in tumors after PDT.

### ΔpO_2_ Correlates with Edema/Erythema

The response to PDT with chlorophyll derivatives was strongly associated with edema and/or erythema. In tumors responsive to PDT, edema sometimes developed immediately after irradiation, usually persisted for several days, and reached its maximum 24 h after PDT. The rapid emergence of interstitial edema around the tumor after PDT might be caused by leakage from vessels [28]. For non-responsive tumors and light control tumors, edema and/or erythema were either absent or short-lived and mild (Fig. 4C). In tumors responsive to PDT, the severity of edema and/or erythema 24 h after irradiation correlated with the percentage increase in partial pressure of oxygen immediately after irradiation (Fig. 4﻿D). The immediate formation of edema around the tumor after PDT may affect pO_2_ in tumor tissue. Edema can alter the rate of blood flow in a tumor and increase the distance for oxygen exchange between vessels and tumor cells [29]. However, the rather rapid intensification and then gradual disappearance of the edema over a longer period of time did not force any significant changes in pO_2_ in the tumors. This suggests that edema does not influence pO_2_. This is also suggested by the results of another study of F_2_BMet PDT in S91 tumors, where strong edema was accompanied by a completely different pO_2_ profile in tumors than in the case of chlorophyll derivatives. After irradiation of tumors with F_2_BMet, edema develops around the tumors, and it is initially accompanied by a decrease in pO_2_, and in the following days, depending on the PDT protocol used, there is either a further long-term decrease in pO_2_ (PDT targeting tumor vessels) or a prolonged increase in pO_2_ (PDT targeting tumor cells) [22]. Effective photodynamic therapy of tumors using chlorophyll derivatives was clearly associated with the development of edema around the tumor. Its maximum intensity (after 24 h) correlates with the relative increase in the partial pressure of oxygen in tumors immediately after irradiation.

### pO_2_ and Superficial Blood Flow

In both control and PDT-responsive tumors, an increase in superficial blood flow was observed during irradiation with the 655 nm laser and was quite similar in both groups (Fig. 5A, B). These results indicate that the irradiation induced reactive hyperemia likely due to tissue heating [24]. Immediately after irradiation, in both control and PDT-responsive tumors, there is a peak in superficial blood flow (Fig. 5), which is associated with a peak in pO_2_ (Fig. 2). This indicates that the increase in blood flow after irradiation is one of the elements determining the increase in partial pressure of oxygen in tumors.

Shortly after PDT, the LDPI signal decreased abruptly due to tissue edema and the subsequent measurement did not reflect the actual blood flow. The signal returned to baseline on day 4, reflecting the decrease in edema (Fig. 5B, C). This indicates that the reduction in the LDPI signal in these tumors is not only due to a decrease in blood flow in the vessels, but also due to the edema of these vessels, which weakens the recorded signal. As the edema gradually subsided, the tumor’s superficial blood vessels were exposed, and therefore, the LDPI signal of superficial blood flow was again better detectable.

In light control tumors, within 24 h of irradiation, surface blood flow decreased to the pre-irradiation level, similarly to pO_2_. In the next 4 days after irradiation, blood flow gradually increased, which probably reflects the gradual development of the vascular network with increasing tumor mass. However, pO_2_ did not change significantly during this time, indicating an oxygen balance between the increasing oxygen consumption of the proliferating tumor cells and the increased efficiency of oxygen delivery by the developing vessels.

## Conclusions

### Is the Peak of pO_2_ the Cause or Consequence of an Effective PDT?

**Fig. 6:**
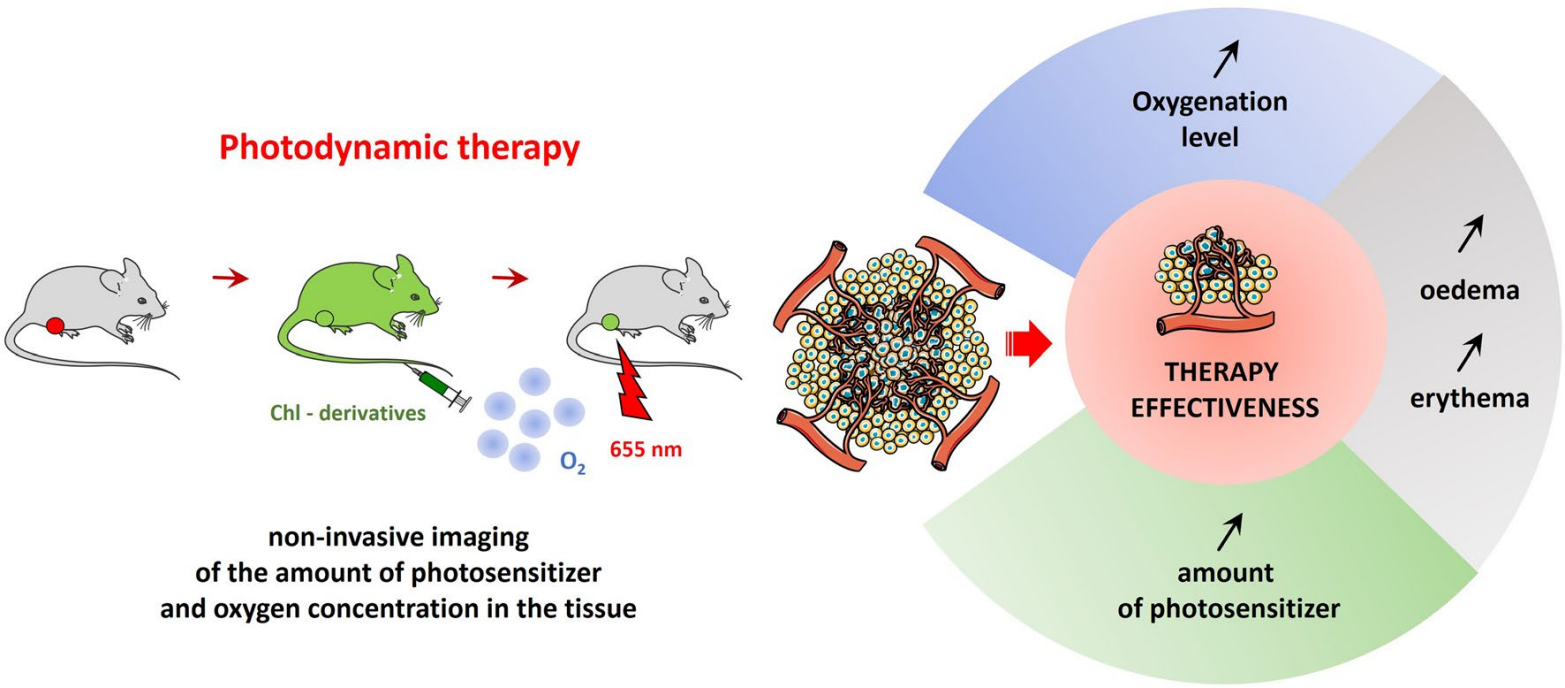
A diagram showing what influences therapy effectiveness. Prognostic factors are the amount of photosensitizer that reached the tumor, ΔpO_2_, and the appearance of severe edema and erythema

The objective of our study was to evaluate whether the monitoring of partial pressure of oxygen (pO_2_) in tumors treated with photodynamic therapy with chlorophyll derivatives can predict treatment response. We have shown that the higher ΔpO_2_ (pO_2_ after PDT minus pO_2_ before PDT), the better the tumor response. The same parameter, ΔpO_2_, correlated with the photosensitizer level in the tumor tissue before PDT and with tissue edema/erythema after PDT (Fig. 6).

The results presented here show that pO_2_ assessment in tumors may be a more widely used predictor of PDT response, but the profile of this correlation must be determined separately for a given class of photosensitizers. In the case of the evaluated metal derivatives of chlorophyll, the more photodynamically effective the compound, the stronger the relative increase in oxygen partial pressure after irradiation. The level of pO_2_ achieved is limited by the rate of oxygen delivery by tumor vessels and the intensity of photodynamic aerobic reactions that lead to its consumption.

### Supplementary Information

Below is the link to the electronic supplementary material. The relationship between pO_2_ and linewidth of the EPR signal of LiPc is close to linear, so the calibration curve was prepared using a linear regression fit to the data points. The resulting line is described by the coefficient value of 5.79 ± 0.42 mG/mmHg and a free expression value of 47.25 ± 5.25 mG (minimum linewidth of the probe signal). The R^2^ fit parameter was 0.98. This line was used in in vivo measurements as a calibration curve to convert the linewidth of the LiPc EPR signal to oxygen partial pressure.

Supplementary file1. Figure S1. Dependence of the signal linewidth of the LiPc probe on partial pressure of oxygen (pO ) inside the capillary. EPR measurements were performed for fixed values of the partial pressures of oxygen, which was obtained by purging the capillary with mixture of argon and oxygen in the proper proportions In the inset, an example of the S-band EPR spectrum of the LiPc probe was presented, together with the indication of the measured peak-to-peak width of the signal. (JPG 173 KB)

Supplementary file2. Figure S2. Chemical structures of Chlide and Zn-Pheide and their representative absorption and emission spectra. (JPG 271 KB)

## Data Availability

The data that support the findings of this study are available from the corresponding author, [M.S], upon reasonable request.
