# Supplementary figures and images for: EPR Monitoring of Oxygenation Levels in Tumors After Chlorophyllide-Based Photodynamic Therapy May Allow for Early Prediction of Treatment Outcome
Source: Mol Imaging Biol. 2024 Jan 31;26(3):411–23. doi: 10.1007/s11307-023-01886-7 (PMC11211189; doi:10.1007/s11307-023-01886-7)

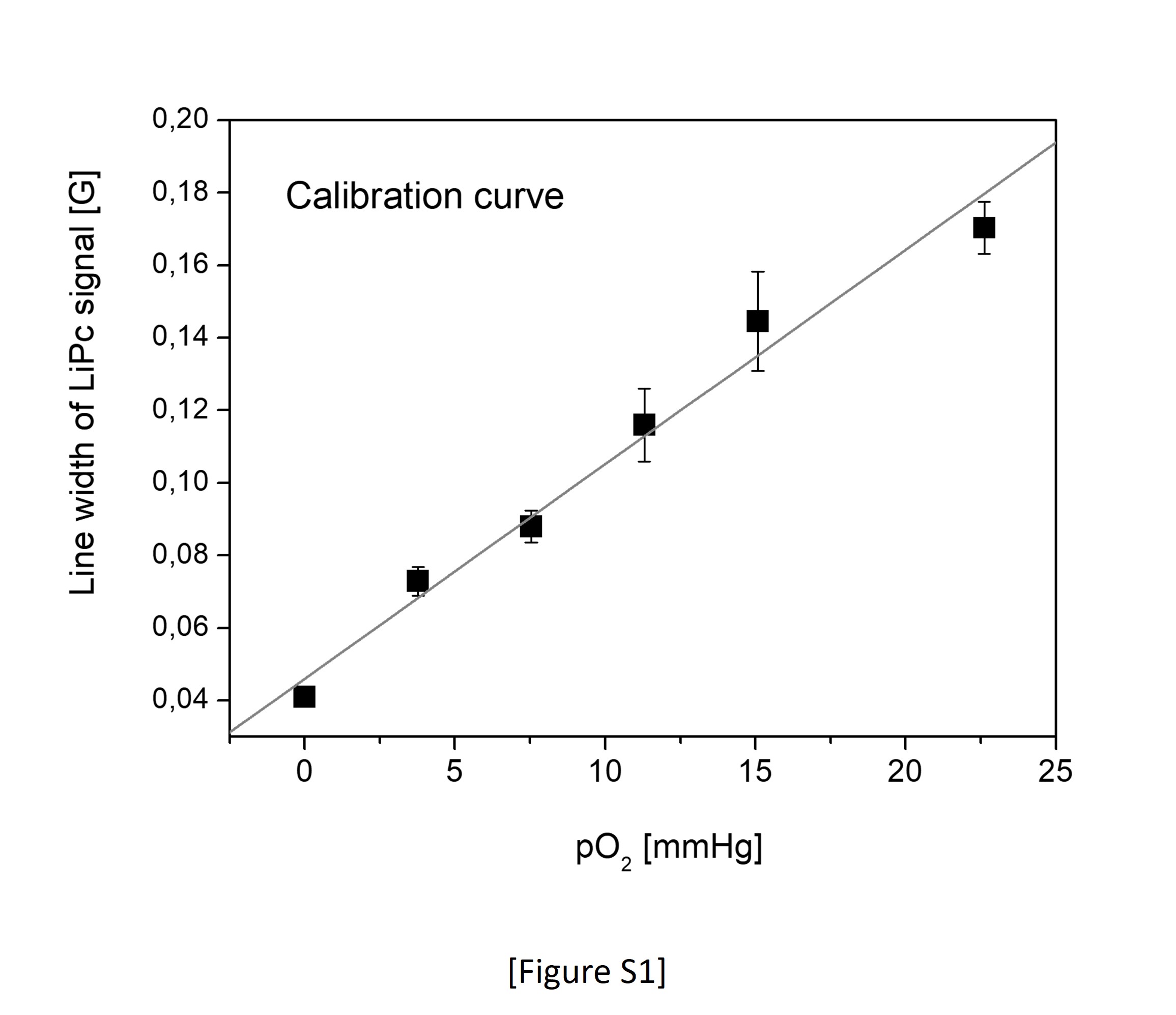

Supplement: Supplementary file 1 — Supplementary file1. Figure S1. Dependence of the signal linewidth of the LiPc probe on partial pressure of oxygen (pO ) inside the capillary. EPR measurements were performed for fixed values of the partial pressures of oxygen, which was obtained by purging the capillary with mixture of argon and oxygen in the proper proportions In the inset, an example of the S-band EPR spectrum of the LiPc probe was presented, together with the indication of the measured peak-to-peak width of the signal. (JPG 173 KB) [file 11307_2023_1886_MOESM1_ESM.jpg]

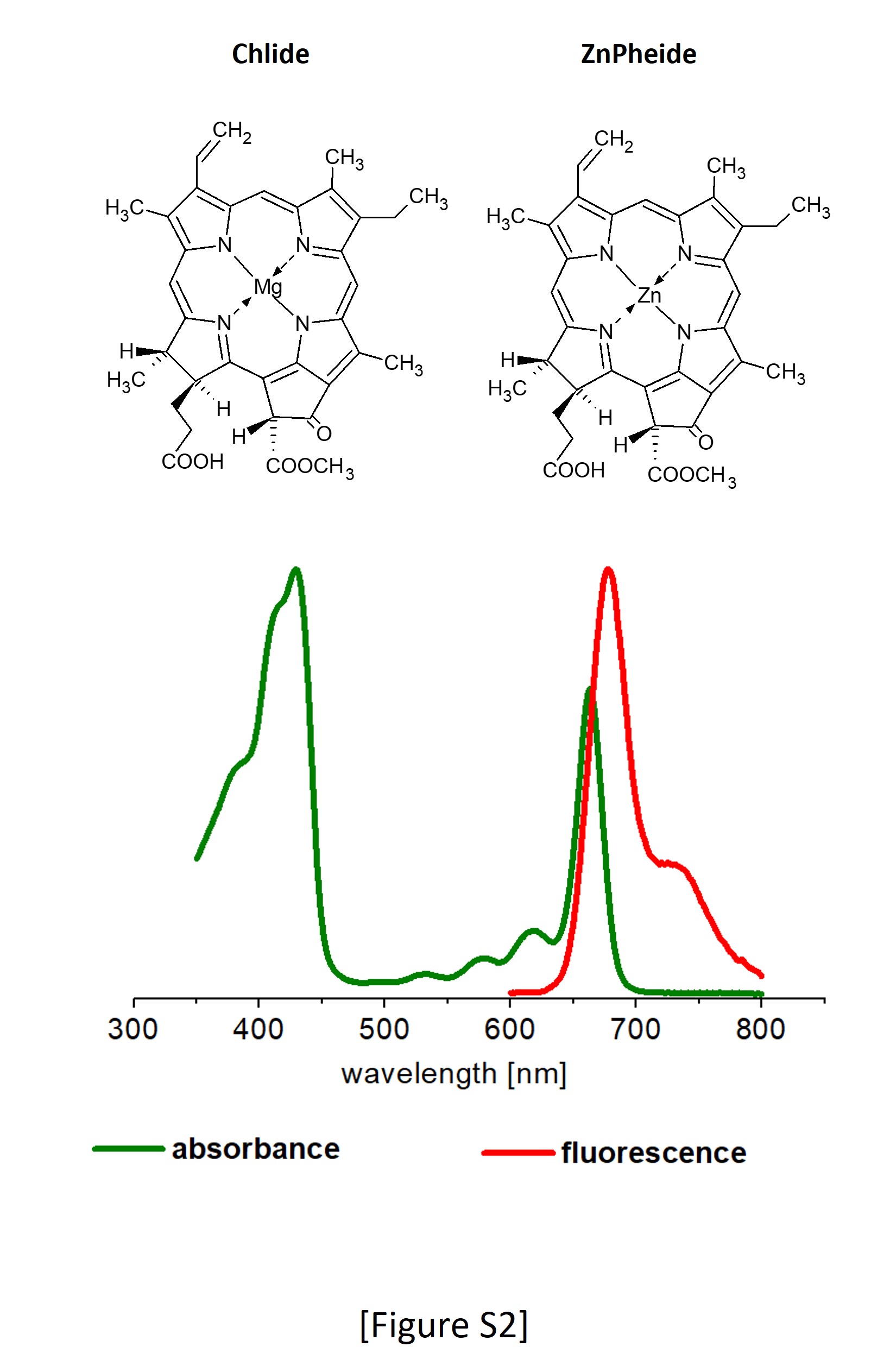

Supplement: Supplementary file 2 — Supplementary file2. Figure S2. Chemical structures of Chlide and Zn-Pheide and their representative absorption and emission spectra. (JPG 271 KB) [file 11307_2023_1886_MOESM2_ESM.jpg]
